# Sequencing and Comparative Analysis of the Straw Mushroom (*Volvariella volvacea*) Genome

**DOI:** 10.1371/journal.pone.0058294

**Published:** 2013-03-19

**Authors:** Dapeng Bao, Ming Gong, Huajun Zheng, Mingjie Chen, Liang Zhang, Hong Wang, Jianping Jiang, Lin Wu, Yongqiang Zhu, Gang Zhu, Yan Zhou, Chuanhua Li, Shengyue Wang, Yan Zhao, Guoping Zhao, Qi Tan

**Affiliations:** 1 National Engineering Research Center of Edible Fungi, Ministry of Science and Technology (MOST), Shanghai, P. R. China; 2 Key Laboratory of Edible Fungi Resources and Utilization (South), Ministry of Agriculture, Shanghai, P. R. China; 3 Institute of Edible Fungi, Shanghai Academy of Agricultural Sciences, Shanghai, P. R. China; 4 Shanghai-MOST Key Laboratory of Health and Disease Genomics, Chinese National Human Genome Center at Shanghai, Shanghai, P. R. China; National Center for Biotechnology Information (NCBI), United States of America

## Abstract

*Volvariella volvacea*, the edible straw mushroom, is a highly nutritious food source that is widely cultivated on a commercial scale in many parts of Asia using agricultural wastes (rice straw, cotton wastes) as growth substrates. However, developments in *V. volvacea* cultivation have been limited due to a low biological efficiency (i.e. conversion of growth substrate to mushroom fruit bodies), sensitivity to low temperatures, and an unclear sexuality pattern that has restricted the breeding of improved strains. We have now sequenced the genome of *V. volvacea* and assembled it into 62 scaffolds with a total genome size of 35.7 megabases (Mb), containing 11,084 predicted gene models. Comparative analyses were performed with the model species in basidiomycete on mating type system, carbohydrate active enzymes, and fungal oxidative lignin enzymes. We also studied transcriptional regulation of the response to low temperature (4°C). We found that the genome of *V. volvacea* has many genes that code for enzymes, which are involved in the degradation of cellulose, hemicellulose, and pectin. The molecular genetics of the mating type system in *V. volvacea* was also found to be similar to the bipolar system in basidiomycetes, suggesting that it is secondary homothallism. Sensitivity to low temperatures could be due to the lack of the initiation of the biosynthesis of unsaturated fatty acids, trehalose and glycogen biosyntheses in this mushroom. Genome sequencing of *V. volvacea* has improved our understanding of the biological characteristics related to the degradation of the cultivating compost consisting of agricultural waste, the sexual reproduction mechanism, and the sensitivity to low temperatures at the molecular level which in turn will enable us to increase the industrial production of this mushroom.

## Introduction

*Volvariella volvacea*, also known as the straw mushroom or Chinese mushroom, is an edible fungus that grows in tropical and subtropical regions. Most artificially cultivated straw mushrooms are produced in China where, in the 18th century, Buddhist monks of Nanhua Temple located in Guangdong Province enriched their diet by developing a primitive method that used fermented paddy straw as the growth substrate. The mushroom was held in such high regard that it was often presented as a tribute to Chinese royalty [1], [2].

In addition to rice straw, *V. volvacea* also grows on water-hyacinth, palm oil bunch wastes, pericarp wastes, banana leaves, and cotton waste [3]. Considered a health food because of its dietary and medicinal attributes [4], the mushroom is popular in southern China, Thailand, Malaysia and the Philippines. Previously ranked fifth among the major commercially-cultivated mushrooms [5], annual production of *V. volvacea* has increased in recent years due to a higher demand for health foods. In 2010, output of the mushroom on the Chinese mainland was 330,000 tons, accounting for more than 80% of global production.

Although *V. volvacea* has been cultivated for ∼300 years, multiple problems associated with the practice has greatly restricted development of the industry. The biological efficiency (conversion of the substrate into mushroom fruit bodies) of *V. volvacea* is only ∼15% on straw-based substrates and 30–40% on cotton-waste ‘composts’ [6], values which are considerably lower compared to other major cultivated species such as *Agaricus bisporus, Lentinula edodes* and *Pleurotus* spp. [3]. Furthermore, *V. volvacea* is a tropical fungus that requires relatively high temperatures (28–35°C) for vegetative growth and fruiting. Moreover, temperatures below 15 °C cause chilling damage to the fruiting body and adversely affect the viability of the fungal mycelia (Figure S1). Routine storage at low temperatures (4 °C) causes fruit body autolysis [7], thereby shortening mushroom shelf-life and hampering distribution over long distances.

**Figure 1:**
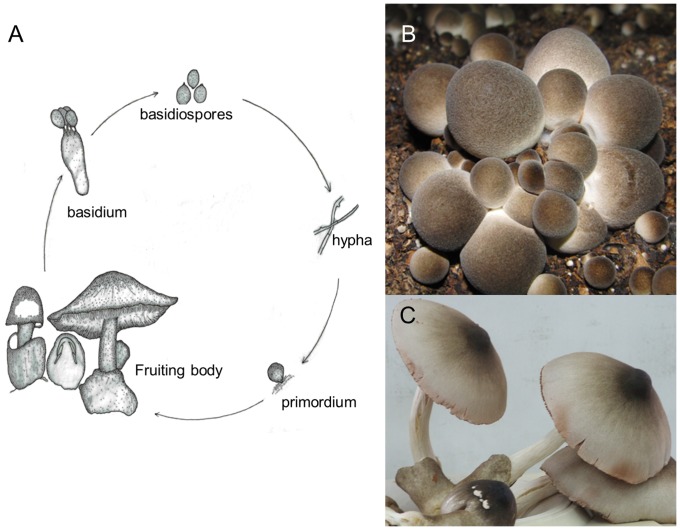
Life cycle and the fruiting body of *Volvariella volvacea*. A. Basidiospores of *V. volvacea* are in tetrad and attached to a basidium by four sterigmata. Mature basidiospore germinates under favorable conditions to form primary mycelium. Secondary mycelia form by the fusion of primary mycelia, and give rise to fruiting bodies which create another new generation of basidiospore. The fruiting mycelium has no clamp connections. The life cycle of *V. volvacea* in general is complete in 4–5 weeks time [11], [90]. B. “Button” stages in the development of *V. volvacea*, which are harvested and sold in the market. C. A mature fruiting body.

*V. volvacea* has been described as a primary homothallic basidiomycete [1], [8], [9], whereby the homokaryotic mycelium arising from the germination of a single basidiospore is able to convert to the dikaryotic form and to complete the sexual cycle without mating (Figure 1). However, *V. volvacea* has multinucleate hyphae [10] and dikaryotic mycelia lack clamp connections, the morphological markers that differentiate the dikaryon from the homokaryon [11].

We now report the complete genome sequence of the monokaryotic *V. volvacea* strain, V23-1. Our data will advance our understanding of the fungal cellulolytic system, thereby facilitating more efficient conversion of the agricultural wastes used as substrates for mushroom cultivation. Our transcript profiles from mycelia exposed to chilling will help identify factors regulating the sensitivity of this tropical fungus to low temperatures. Further research based on our results should also lead to recognition of the genetic determinants controlling sexuality in *V. volvacea* and the breeding of improved mushroom strains.

## Results and Discussion

### Genome sequencing and general features

The *V. volvacea* genome was sequenced using Roche 454 GS FLX and Illumina Solexa sequencing technologies. Combined sequences from the two technologies generated 190×coverage of the genome, and were assembled into 62 scaffolds with an N50 of 388 kb and a total genome size of 35.7 Mb (Table 1). The *V. volvacea* genome is similar in size to the genomes of several other species from the order Agaricales, including *Schizophyllum commune* (38.5 Mb) [12], *Coprinopsis cinerea* (37 Mb) [13] and *P. ostreatus* (35 Mb) [14], but larger than that of *A. bisporus* (30.2 Mb) [15].

**Table 1:** Features of the *V. volvacea* genome

General features	
Size of assembled genome (Mb)	36.45
GC content (%)	48.86%
Length of classified repeats (%)	2.25 Mb (6.18%)
Number of predicted gene models	11,084
Average gene length (with intron) (bp)	2,087
Average transcript length (bp)	1,572
Number of single-exon genes	1,066
Average number of exons per multi-exon gene	7
Average exon size (bp)	229
Average intron size (bp)	88
Number of transposase-related genes	12
Number of tRNA genes	158
**Properties of predicted gene models**	
NR alignment	9,240
KEGG alignment	9,247
KOG assignment	4,821
KO assignment	2,831
GO assignment	5,161
InterPro signature	5,904
Signal peptide	824
Transmembrane domain	1,681

Annotation of the assembled genome sequence generated 11,084 gene models, 76.43% of which are supported by Expressed Sequence Tag (EST) data. Approximately 9,370 (84.5%) gene models were assigned putative biological functions and the remaining 1,714 were hypothetical proteins and are presumed to be *V. volvacea* specific genes. Among the 11,084 gene models, 3,819 (34.5%), 1,995 (18.0%) and 1,617 (14.6%) were homologous to genes in *Laccaria bicolor*, *C. cinerea* and *Serpula lacrymans* var. *lacrymans*, respectively.

The average transcript length was 1,572 bp. Each gene contained an average of seven exons, with an average size of 229 bp, and introns with an average size of 88 bp (Table 1). Totally, 9,240 (83.4%) genes encoded proteins with homologous sequences in the NCBI nr protein databases, and 9,247 (83.4%) genes are mappable through the KEGG pathway database (Table 1).

An InterproScan analysis identified 2,103 conserved protein families in *V. volvacea* (containing 5,904 proteins) (Table S1), and KEGG analysis revealed 2,831 proteins involved in different pathways (Table S2). GO analysis assigned 5,161 proteins into different GO terms (Table S3). Altogether, 4,329 proteins were assigned to different KOG classes, and 2,518 protein domains were revealed in a total of 6,852 proteins (Table S4).

The genome encodes 824 secreted proteins and 1,681 transmembrane proteins (Table 1), indicating that 22.6% of encoded proteins are associated with the extracellular environment. The number of transfer RNA (tRNA) units was estimated to be 158 (Table S5). We observed 304 repeat units, with the total length of 2.25 Mb and occupying 6.18% of the entire genome, and 2,065 microsatellites (Table S6).

We observed a higher number of proteases (261) compared to other straw-rotting mushrooms such as *A. bisporus* (243) and *C. cinerea* (247) (Table S7). However, the number of protease families in these straw-rotting mushrooms (average 250 genes) was significantly smaller compared to the phytopathogenic fungi, *Fusarium graminearum*, *Magnaporthe oryzae*, *Botrytis cinerea* and *Sclerotinia sclerotiorum* (average 350 genes) and insect pathogenic fungi, *Cordyceps militaris*, *Metarhizium anisopliae* and *M. acridum* (average 390 genes) [16]. Representatives of the S01 trypsin subfamily, generally recognized to be virulence factors and pathogenicity markers [17], were absent in all straw-rotting mushrooms, suggesting that trypsin-like proteinases are not necessary for saprophytes [18]. The number of S08 subtilisin subfamily members, involved in the degradation of host cuticles and insect pathogenic fungal infections, was also smaller (11 on average among *V. volvacea*, *A. bisporus*, *C. cinerea*, and *S. commune* compared with 30 in insect pathogenic fungi). The number of M43 metallopeptidase subfamily members was significantly higher in *V. volvacea* (24) and *C. cinerea* (28) compared to just one in both phytopathogenic and insect pathogenic fungi [16]. Twenty-four *V. volvacea* M43 metallopeptidase genes were homologs of the *P. ostreatus PoFE* gene (E value<1.10e^−22^) expressed in mycelia [19] and the *PoMTP* gene (E value<2e^−36^) expressed in fruit bodies [20]. *PoFE* encodes a fibrinolytic enzyme with applicaton in thrombolytic therapy [19] while PoMTP plays an important role in the initiation and formation of mushroom fruit bodies [20].

Cytochrome P450 (CYP) plays various roles in fungi, including detoxification, degradation of xenobiotics and biosynthesis of secondary metabolites [21]. Similar to other basidiomycetes an average of 138 CYPs was observed in *V. volvacea* (Table S8). However, *V. volvacea* has the highest relative number of CYPs (1.24% of the total gene models) compared to other closely related mushroom species such as *C. cinerea* (1.00%), *A. bisporus* (1.11%) and *S. commune* (0.86%) (Table S8). Size of the fungal P450 could indicate an evolutionary history and adaptation to the environment [21]. CYP5136, CYP662, CYP665 and CYP 666 were present only in *V. volvacea* suggesting that the P450 family expanded in this fungus to meet the metabolic demand for biodegradation in various ecological niches (Table S9). Several fungal CYPs (i.e. CYP 58, 59, 60, 64, 65, 68, 505, 526), reported to be involved in the biosynthesis of aflatoxins, trichothecenes, and funonisins [22], [23], [24], [25] were not detected in *V. volvacea*. Other CYPs apparently lacking in *V. volvacea* and in some other basidiomycetes include CYP 56, essential for the formation of the outer spore wall layer in *Saccharomyces cerevisiae* and *Candida albicans* [26], [27], [28], (possibly indicating divergent biosynthetic pathway for spore wall formation in yeast and mushroom species), and CYP505, involved in fatty acid metabolism in *Fusarium oxysporum* [29], [30] (Table S9).

**Figure 2:**
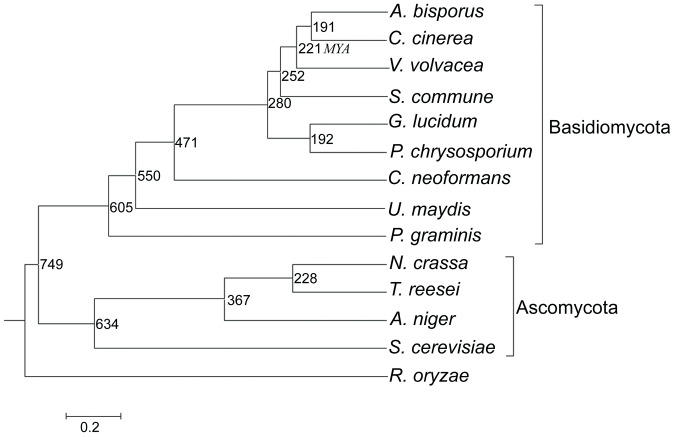
Phylogenomic tree showing the evolutionary distance of *V. volvacea* with different fungal species. *Rhizopus oryzae* (zygomycete) was used as an outgroup. The topology of the phylogenomic tree was constructed by the neighbor-joining method [85] (bootstrap  = 1000, JTT matrix). All bootstrap values are 100%. Time scale was showed by MYA (million years ago). The divergence time of species is located on the right side of each node. The average deviation of divergence time for each node time is only 0.23 MYA.

Phylogenomic analysis juxtaposing *V. volvacea* and 13 other fungi revealed closest evolutionary affinity with *A. bisporus* and *C. cinerea* (divergence time of 221 million years ago [MYA]) and *S. commune* (252 MYA) (Figure 2). The number of reductions in the *V. volvacea* gene families was 907, higher than the average number in other straw rotting fungi (567) and wood decay fungi (399) (Figure S2). This indicates that reduction in number of genes occurred during the evolution of *V. volvacea*. Interestingly, five large gene families (>160 genes) (in a total of 1,154 genes) were present in the *V. volvacea* genome, compared with 2 (452 genes) in *A. bisporus*, 3 (570 genes) in *C. cinerea* and 2 (655 genes) in *S. commune* (Figure S3). We suggest that functions of some gene families in *V. volvacea* may be enhanced during evolution for adaptation to specific growing niche. Furthermore, on a genomic scale, the percentage of amino acid sequence similarity was greater than 80% in only 257 pairs proteins in *V. volvacea*, virtually equal to the 255 pairs in *C. cinerea*, but much lower than 468 pairs in *A. bisporus* and 383 pairs in *S.commune* (Figure S4).

### Mating type locus and sexuality pattern

**Figure 3:**
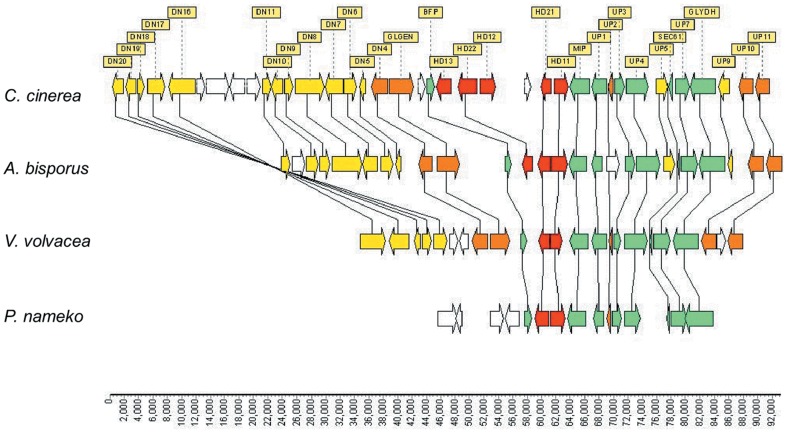
Comparison of the genomic structure of *A* mating type locus and its flanking region among *C. cinerea*, *A. bisporus*, *V. volvacea* and *P. nameko*. Homologous genes are connected with lines. Arrows indicate the putative direction of transcription. Homeodomain protein genes (HD genes) are shown as red boxes. Green boxes indicate genes that are conserved among four species. Orange boxes indicate genes that are conserved among three species. Yellow boxes indicate genes that are conserved between two species. White boxes indicate genes that are not conserved among the four species. Data stem from *C*. *cinerea* (positions 2602748-2694125 from NCBI GenBank accession no. AACS02000001), *A*. *bisporus* (positions 862530-932075 from scaffold 1 in JGI *Agaricus bisporus* genome sequence, http://genome.jgi.doe.gov/Agabi_varbisH97_2/Agabi_varbisH97_2.home.html), *V. volvacea* (positions 379806-432904 from scaffold 7, HD1 position 406228–407814 and HD2 position 406060–404643) and *P*. *nameko* (positions 1734–39882 from NCBI GenBank accession no. AB435542).

A Blastx scan of the *V*. *volvacea* V23-1 genome using basidiomycete homeodomain (HD) genes as the query, identified VVO_04854 and VVO_05004 genes (designated *vv-HD1* ^−V23−1^ and *vv-HD2* ^−V23−1^) located 271 bp apart on scaffold 07. Both encoded HD protein exhibiting a high similarity to homologs in the bipolar basidiomycetes, *A. bisporus* and *Pholiota nameko*, and tetrapolar basidiomycetes, *C. cinerea* and *S. commune*. It appears that *V*. *volvacea* has a single *A* mating type locus composed of two HD genes. Comparison of the genomic region flanking the *V*. *volvacea A* mating type locus with corresponding regions in *A*. *bisporus*, *P*. *nameko* and *C*. *cinerea* revealed a highly conserved synteny (Figure 3). When a pair of specific primers (VVmipF11 and VVmipR13), based on the sequence surrounding the mating type *A* locus in strain V23-1 (*A*1 locus), were used to amplify the *A* locus of the single-spore-isolate V23-18 (*A*2 locus), two genes, *vv-HD1-* ^V23−18^ and *vv-HD2*-^V23−18^ (Accession number: JX157875) were identified within the *A*2 locus. These encoded HD1 and HD2 proteins that were 48% and 49% similar to the corresponding proteins from V23-1.

Three genes, VVO_01536, VVO_09012 and VVO_09031 (designated *vv-rcb1*, *vv-rcb2*, *vv-rcb3*, respectively), located 72 and 4 kb apart on scaffold 24, were identified by Blast searches aimed at recognizing pheromone-receptor-like gene homologs. Deduced protein sequences of *vv-rcb1*, *vv-rcb2* and *vv-rcb3* genes were similar to other pheromone receptors and contained 7-transmembrane structures, which are characteristic of pheromone receptors in basidiomycetes with a mating type *B* locus [31]. However, although these data indicated a mating type *B* locus, the sequences of the putative locus and flanking region showed poor synteny when compared to other basidiomycetes. Furthermore, the genes encoding the pheromone precursor, which is required for the functioning of *B* mating factor [31], were not evident in the flanking region of the *vv-rcb1*, *vv-rcb2* and *vv-rcb3* genes. The DNA sequences of a second set of three pheromone-receptor-like genes, amplified using specific primes from a putative mating type *B* locus in strain V23-18 (which is compatible with V23-1), were identical to corresponding sequences in V23-1. Therefore, pheromone-receptor-like genes in *V*. *volvacea* may not regulate mating compatibility in the same way as in the bipolar fungi *Coprinellus disseminatus* [32] and *P*. *nameko* [33].

Molecular markers for *A* mating type genes were identified using two pairs of gene-specific primers (A1168F and A1168R for the *A1* locus, and A2217F and A2217R for the *A2* locus) designed according to the allelic sequences in mating type *A* locus. These markers can be used to distinguish homokaryotic and heterokaryotic single spore isolates (SSIs), and more effective than microscopic observations or SCAR (Sequenced Characterized Amplified Region) markers. A total of 124 SSIs derived from strain V23 were separated into three groups based on the presence of *A1* and/or *A2* locus as follows: A1 group (35 SSIs, 28.2%), A2 group (66 SSIs, 53.2%) and A1A2 group (23 SSIs, 18.6%). The 18.6% incidence of heterokaryons is higher than found among SSIs derived from *V. volvacea* strain Pingyou No. 1 (7.14%) [34] but considerably lower than reported among SSIs taken from strains H (77%, 23/30) and K (75%, 15/30) [9]. Long term artificial cultivation and/or a bigger test sample may account for the low incidence of heterokaryons among SSIs strains V23 and Pingyou No. 1. Nevertheless, the presence of both homokaryon and heterokaryon among SSIs derived from V23 is entirely consistent with previous observations [9], [34]. Furthermore, in accordance with earlier data obtained with the H and K strains of *V. volvacea* [9], cultivation tests undertaken in this study involving 12 SSIs assigned to the A1A2 groups and 14 designated homokaryotic SSIs confirmed that only the former were able to fruit. Of eight heterokaryons obtained by crossing SSIs belonging to the A1 and A2 groups, seven produced fruit bodies, indicating that the *A* mating type genes in *V. volvacea* regulate mating compatibility as in other mushrooms exhibiting bipolar sexual reproduction.

**Figure 4:**
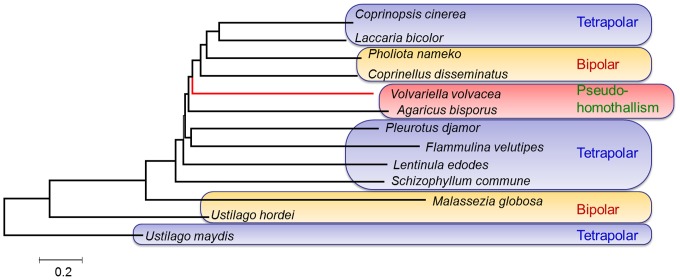
Phylogenetic tree constructed from the amino acid sequence alignments of homeodomain mating-type protein homologs of *V. volvacea* and various basidiomycete fungi.

Phylogenetic analysis suggests that the bipolar HD proteins encoded by the *A* mating type genes in *V. volvacea* and other basidiomycetes originated from an ancestral HD protein associated with the tetrapolar basidiomycete, *Ustilago maydis* (Figure 4), supporting earlier claims [35], [36] that bipolar systems evolved from tetrapolar counterparts. The phylogenetic tree also indicated a close relationship between *V. volvacea*, *A. bisporus*, *C*. *disseminatus* and *P*. *nameko*. Loss of the *B* mating factor function in *V. volvacea* correlates with the inactivation of pheromone receptor-like genes [32], [37].

Although *V. volvacea* has often been described as primary homothallic, this contention is still the subject of debate [38], [39], [40]. The phylogenetic data presented here support the notion that *V. volvacea*, like *A. bisporus* [41], is pseudo-homothallic. Such a system decreases the probability of out-breeding and increases the potential for inbreeding. This does not encourage genetic polymorphism, but ensures the high frequency of self-fertility and fruiting for effective transfer of genes required to flourish in a particular ecological niche (e.g. 30–35°C).

### Carbohydrate active enzymes (CAZymes)

**Figure 5:**
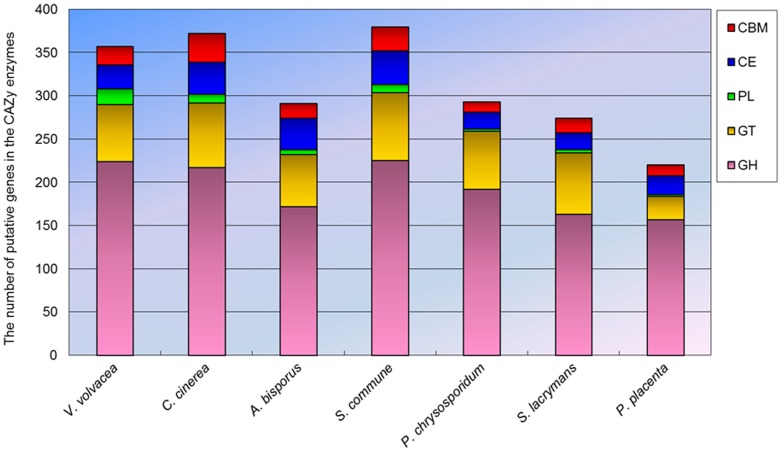
Comparison of the number of five CAZy genes in *V. volvacea* and other basidiomycetes fungi genomes.

**Figure 6:**
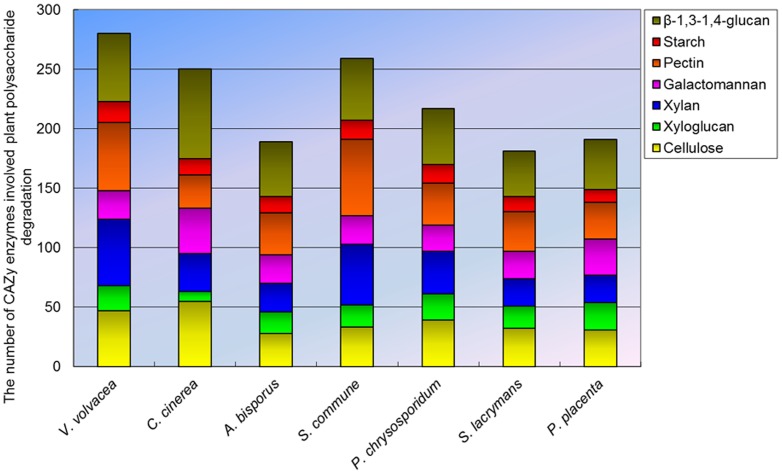
Number of putative enzyme models involved in plant cell-wall degradation in *V. volvacea* and other basidiomycetes fungi genomes.

A total of 357 CAZyme-coding gene homologs were identified in the genome of *V. volvacea* more than the average (305) reported for several other basidiomycetes (Table S10). Of these, 224 homologs belong to the glycoside hydrolases (GH) superfamily, and respresented 42 families. Carbohydrate esterase (CE), glycosyl transferase (GT), polysaccharide lyase (PL) and carbohydrate-binding module (CBM) superfamilies were represented by 28, 66, 18 and 21 homologs respectively, distributed among 8, 24, 3 and 6 families, respectively (Figure 5, Table S10). The 18 PL models was the highest number recorded among several basidiomycetes. Analysis of CAZy families involved in plant polysaccharide degradation revealed 47, 101, and 57 candidate CAZymes related to the degradation of cellulose, hemicellulose and pectin, respectively in *V. volvacea* compared with the corresponding average values of 36, 77 and 37 for the other basidiomycetes (Figure 6, Table S11). *V. volvacea* also contained the highest number of putative β-1, 4-endoglucanases assigned to family GH 7 (14 compared with an average of 4 for other basidiomycetes), putative β-1,4-endoxylanases [42] belonging to family GH 10 (19, average 4), and putative pectin and pectate lyase (PL1) [42] (11, average 1). Interestingly, 30 putative GH 61 genes were detected in the genome, which is higher than the average found in other straw rotting fungi (*C. cinerea* and *A. bisporus*) (23), white rotting fungi (*S. commune* and *P. chrysosporium*) (18) and brown rotting fungi (*P. placenta* and *S. lacrymans*) (5). Recently, several GH 61 proteins have been shown to increase the activity of cellulases during lignocellulose degradation [43], [44]. The *V. volvacea* genome also contained a relatively large number of genes (57) encoding enzymes involved in the degradation of β-1,3-1,4-glucan, which are widely distributed as non-cellulosic matrix phase polysaccharides in cell walls of grasses and cereal species.

### Fungal oxidative lignin enzymes

Although *V. volvacea* is generally regarded as a straw-degrading fungus, low substrate biological conversion rate have been attributed to an apparent lack of an efficient ligninolytic enzyme system [45], [46], [47]. Ligninolytic fungi degrade lignin using combinations of multiple isoenzymes of three heme peroxidases: lignin peroxidases (LiPs), manganese peroxidases (MnPs), and hybrid enzymes known as versatile peroxidases (VPs). The *V. volvacea* genome contains two putative MnP- and two putative VP-encoding genes, but lacks genes encoding LiPs (Table S12). In addition, 11 genes (*vv-lac1* to *vv-lac 11*) encoding putative laccases, enzyme involved in various biological processes including lignin degradation, fungal development, melanin synthesis, and fungal pathogenesis of human and plant hosts [48], were also identified (Table S13). Six of these were identical to laccase genes isolated (by PCR using degenerate primers) in previous study [49], [50] (Table S13). The genomes of other straw-degrading mushrooms such as *A. bisporus* (12) and *C. cinerea* (17) also contain multiple laccase genes (Table S12). Ten of the eleven laccase genes in the *V. volvacea* genome were distributed through a 216 kb region in scaffold 6, and this gene cluster is supported by hypergeometric analysis. With the exception of *vv-lac* 11, all the other putative laccase genes were (when compared to *A. bisporus* and *C. cinerea*) clustered within a relatively short chromosomal region (Figure S5). *Vv-lac 11* was located in scaffold 8 and closely resembles *vv-lac6*, possibly indicating a paralogous relationship (Figure S6A and Figure S6B). Phylogenetic analyses revealed that nine laccase genes (*vv-lac1-5* and *vv-lac7–10*) are in the same cluster (Figure S6A). The locations of 17 out of 19 introns present in these nine laccase genes are conserved (Figure S6B), whereas, in *C. cinerea* the locations of only two out of 16 introns are conserved [51]. This might imply that the laccase genes in *V. volvacea* have derived from independent duplication-divergence events after speciation [52].

Although the functionality of individual laccase has yet to be confirmed, Chen et al. reported a significant increase in the expression of *lac4* (*vv-lac3* in this study) during pinhead formation, the first stage of fruit body development [50].

### Transcriptional analyses of cold shock response

**Figure 7:**
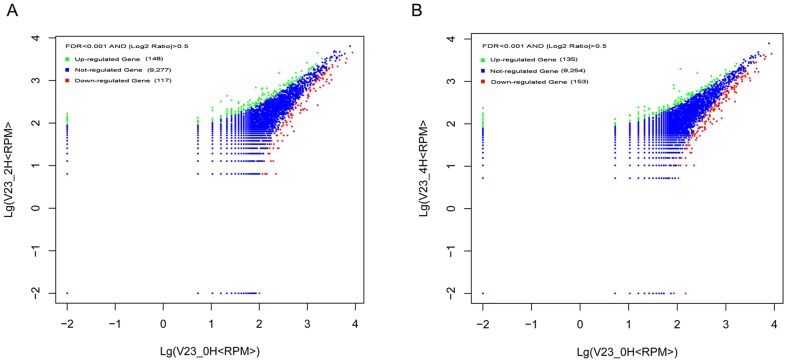
Differential gene expression in *V. volvacea* after exposure to 4 °C for different times. Comparison of differentially expressed genes between 0 and 2 h (A), 0 and 4 h (B).

*V. volvacea* is unable to tolerate chilling injury resulting from contact with lower temperatures (above freezing), and exposure to 4 °C for more than eight hours causes irreversible damage leading to loss of viability [7]. High-throughput sequencing of mRNA expression in the vegetative hyphae exposed to 4 °C for 0, 2 and 4 h yielded 188999, 156430 and 191607 reads, respectively. Normalization of the data using DEGseq software disclosed that all the expressed genes participated in at least one of 270 metabolic pathways (Table S14). Comparison of the differences in gene expression levels between zero and 2 h, and zero and 4 h, using│log_2_ (fold-change)│> = 0.5 and FDR<0.001 as the threshold for significant changes, revealing 148 and 135 genes were up-regulated at 2 h and 4 h, respectively. The corresponding numbers of down-regulated genes were 117 and 153, respectively (Figure 7A, Figure 7B, and Table S15). Interestingly, 55 genes were both down-regulated at 2 and 4 h compared with only 25 up-regulated genes (Table S15). Among the 25 up-regulated genes, 19 were found to be conserved hypothetical proteins. VVO_05539 gene (log_2_ (0 H/2 H fold-change)  = 1.68, FDR = 0.0004; log_2_ (0 H/4 H fold-change) = 1.89, FDR = 5.77E^−06^) encodes a putative protein containing an F-box domain for protein-protein interaction that confers substrate specificity for ubiquitination. VVO_00268 gene (2.52, 1.19E^−34^; 2.60, 1.19E^−41^) encodes a putative protein with the conserved domain for the ubiquitin-conjugating enzyme E2. The inferred amino acid sequence of VVO_05394 (2.10, 4.51E^−09^; 4.07, 7.68E^−77^) gene shows a 71% identity and a 76% similarity with a senescence-associated protein in *Picea abies* (E value: 4e^−56^). Senescence-associated genes have generally been found to be up-regulated during leaf senescence, and the senescence-associated proteins such as RNases, proteinases, and lipases function as degradation enzymes [53], [54]. The inferred amino acid sequence of VVO_06566 (2.73, 0.00097; 3.27, 1.21E^−06^) gene contains the conserved domain for HNH (His-Asn-His) nucleases.

Among the 55 down-regulated genes at 2 and 4 h, 28 encode conserved hypothetical proteins. Eight of these (VVO_02676, VVO_07414, VVO_05796, VVO_05198, VVO_05208, VVO_09166, VVO_09167 and VVO_10542) are related to heat shock proteins (HSP) (Table S16). HSPs are reported to play an important role in protein folding when proteins are denatured due to oxidative stress, high pressure, and heat shock [55], [56], [57]. A number of HSP genes in *Saccharomyces cerevisiae* have been found to be induced between 6–48 h after exposure to temperatures as low as 4 °C [58]. HSPs in *V. volvacea* were down-regulated after exposure to 4 °C, although it remains unclear if this is connected to the higher sensitivity of the mushroom to lower temperatures.

Genes involved in trehalose and glycogen biosynthesis in yeast are induced after exposure to 4 °C [57], [59], suggesting that biosynthesis and accumulation of the reserve carbohydrates may be linked to cold tolerance and energy preservation [57]. Expression analysis of genes involved in starch and sucrose metabolism pathways (PATH: ko00500) revealed that 32 such genes were expressed at 0, 2 and 4 h following exposure to 4 °C (Figure S7, Table S17), although expression levels of the majority exhibited no significant change. Among them, VVO_02898 and VVO_08127, genes which encode the homologs of TPS 1 (trehalose-phosphate synthase) and TPS 2 (trehalose-6-phosphate phosphatase) in yeast, were repressed (−2.032, 9.01E^−8^) and no significant change at 2 h after exposure to 4°C. However, expression levels of TPS 1 and TPS 2 genes in *S. cerevisiae* were increased 5.6- and 5.9-fold respectively at 0°C [58], and 2- and 2.6-fold respectively at 4°C [57]. Genes involved in the glycolysis and gluconeogenesis pathways (PATH: ko00010) in *V. volvacea* was either repressed or showed no significant increase in expression after 2 and 4 hours exposure to 4°C (Figure S8, Table S18). These included VVO_06963 (encoding glyceraldehyde 3-phosphate dehydrogenase), VVO_03893 (encoding phosphoenolpyruvate carboxylkinase) and VVO_09324 (encoding aldehyde dehydrogenase family 7 member A1). Similarity, no significant increase in the expression of VVO_01210 (encoding stearoyl-CoA desaturase), VVO_00398 (encoding omega-6 fatty acid desaturase) and VVO_10684 (encoding acetyl-CoA acyltransferase 1), key genes involved in the biosynthesis of unsaturated fatty acids (PATH: ko01040), was observed following exposure to 4°C (Figure S9, Table S19). However, VVO_00127 (encoding 3-oxoacyl-[acyl-carrier protein] reductase, FabG) was expressed at 2 h (log_2_ (fold change) = 2.48, FDR = 7.22E^−14^), but not at 4 h (log_2_ (fold change) = 0.40, FDR = 0.56).

Although a variety of life forms indigenous to the tropics are unable to tolerate low temperatures, the underlying mechanisms are not well understood [60]. Exposure to low temperatures often triggers a common cold-shock response involving the biosynthesis of unsaturated fatty acids, accumulation of trehalose and glycogen, and the appearance of cold shock and heat shock proteins [61]. However, Li *et al* (1992) reported a significant decrease in the levels of unsaturation in *V. volvacea* membrane lipids at 0°C [62] and, while analysis of the *V. volvacea* genome revealed the presence of genes encoding enzymes involved in unsaturated fatty acids, trehalose and glycogen biosynthetic, the levels of gene over-expression recorded elsewhere was not evident. Thus, while biochemical adaptations and mechanisms that regulate low temperatures tolerance elsewhere have been described [61], these have yet to be elucidated in *V. volvacea*.

Sequencing of the *V. volvacea* genome provides a model for studying the evolution of specific molecular mechanisms associated with the saprophytic mode of nutrition, low temperature sensitivity and mating pattern exhibited by *V. volvacea*. Our data will serve as an information platform for future research directed at improved industrial production of this economically important edible mushroom.

## Materials and Methods

### Strains and culture conditions

The *Volvariella volvacea* dikaryotic strain, V23, which is widely cultivated in China, was provided by the Institute of Edible Fungi, Shanghai Academy of Agricultural Sciences. Twenty single spore isolates were obtained by spreading a suspension of basidiospores of strain V23 on Potato Dextrose Agar (PDA) plates (Becton, Dickinson, Sparks, USA) and confirmed as pure homokaryons by an inability to form sporophores under conditions that promoted fruit body formation by the dikaryotic parent. One of these, V23-1, was selected at random and used for genome sequencing.

### Amplification of genes within the *A* mating type locus

The specific primer pair, VVmipF11 (5′-GTGACTGCTATGGAACACATTGGAC) and VVmipR13 (5′- TCGGAGGAAGCGGGTCCACTACA), was used to amplify the *A* mating type locus of *V. volvacea*. The two specific primer pairs, A1168F (5′-AGGGCATTCCAACCTATTCGCTTTC) and A1168R (5′-AATGTGAACAGTTTGAGCGGAGT), and A2217F (5′-GTGGTTGGGATGGAAGGTTGTGA) and A2217R (5′-CTGTGAGGGTTTGTGGTGGGATA), were used to amplify the *A1* and *A2* loci, respectively.

### DNA extraction, genome sequencing, assembly and annotation

Genomic DNA was extracted from fungal mycelium using an improved cetyltrimethylammonium bromide (CTAB) method [63] as follows: freeze-dried mycelia (100 mg) was ground into a powder using a mortar and pestle and mixed with 1 mL CTAB buffer [2% CTAB, 1.4 M NaC1, 100 mM Tris HC1 (pH 8.0), 20 mM EDTA (pH 8.0)] preheated to 65°C. The homogenate was centrifuged (9650 g, 20 min, room temperature) and an equal volume of chloroform:isoamyl alcohol (24 1) added to the retained supernatant fraction. After gentle mixing, the suspension was kept at room temperature for 15 min and then centrifuged as above. The supernatant was transferred to a new microtube containing two-thirds volume of cold (−20°C) isopropanol and, after gentle shaking, the mixture was centrifuged (6450 g, 10 min, room temperature). The DNA pellet was washed three times with 1 mL 75% (v/v) ethanol (containing 10 mM potassium acetate), once with 1 mL cold 95% (v/v) ethanol, and air-dried. After dissolving the pellet in 50 µL TE buffer, RNA was removed by adding 1 µL RNase solution (10 mg/mL) and incubating at 37°C for 1 h. The DNA solution was then stored at −20°C until use.

The genomic DNA was sequenced using the Roche 454 GS FLX (Roche, USA) and Illumina Solexa GAIIx (Illumina, USA) platforms. Following pre-processing, the Roche 454 reads were assembled into a primary assembly using the Roche Newbler software and then scaffolds were constructed with Illumina paired-end and mate-pair reads using Velvet software (1.2.03) [64]. Gene model prediction was performed by combining four sets of software: GeneMark (2.8) [65], Augustus [66], FGENESH (2.6) [67] and GeneID (1.4.4) [68].The final gene model was determined with “GLAD”, an in-house developed automatic validation program that validated the final gene model employing gene length, EST frequency and annotation data. RepeatScout [69] and SciRoKo 3.4 software [70] was used to identify repetitive sequences and microsatellite sites in the genome, respectively.

### RNA extraction, mRNA **Purification,** cDNA Synthesis, cDNA Sequencing

Fungal mycelium of *V. volvacea* strain V23 was grown in 250 mL flasks containing 100 mL Potato Dextrose Broth (PDB) (Becton, Dickinson, Sparks, USA) at 32 °C for 4 days, and then exposed to 4 °C for 0, 2 and 4 h. The treated mycelia were collected and lyophilized, and total RNA was extracted using Trizol reagent (Invitrogen, Carlsbad, USA) according to manufacturer's instructions. Double-stranded cDNA was synthesized from mRNA using the method of Ng et al with modifications [71]. A GsuI-oligodT primer and 1000 units of Superscript II reverse transcriptase (Invitrogen, Carlsbad, USA) were used to synthesize first-strand cDNA from 10 µg of mRNA. After incubation at 42 °C for 1 h, the 5′-CAP structure of mRNA was oxidized with NaIO_4_ (Sigma, USA) and ligated to biotin hydrazide. Biotinylated mRNA/cDNA was selected by binding to Dynal M280 beads (Invitrogen, Carlsbad, USA). After second strand cDNA synthesis, the polyA tail and 5′ adaptor were removed by GsuI digestion.

cDNA was size-calibrated using a cDNA size fractionation column (Agencourt), and fractions larger than 800 bp were sonicated to between 300–800 bp. These were then pooled together with other cDNA samples within the same size range, and transformed into single-stranded template DNA (sstDNA) libraries using the GS DNA Library Preparation kit (Roche Applied Science, USA). Libraries were clonally amplified in a bead-immobilized form using the GS emPCR kit (Roche Applied Science, USA) and sequenced with a 454 Genome Sequencer FLX system.

### Identification of differentially expressed genes

The reads of three treated samples were separately mapped to predicted *V. volvacea* genes using BLASTN, and the expressed reads number of each gene was transformed into RPKM (Reads Per Kilobase per Million mapped reads) [72]. Differentially expressed genes were identified with the DEGseq package using the MARS (MA-plot-based method with Random Sampling) method [73]. A FDR ≤0.001 and an absolute value of log_2_Ratio ≥0.5 were adopted as threshold levels to assess the significance of contig expression difference.

### Protein family classification and family size analysis

Whole genome protein families were classified by InterProScan [74] (http://www.ebi.ac.uk/interpro/) analysis and Pfam 26.0 [75] (http://pfam.sanger.ac.uk/) analysis. Protease families were additionally classified using Blastp against the MEROPS 9.7 peptidase database (http://merops.sanger.ac.uk/) [76]. Cytochrome P450s were named according to the P450 database (http://drnelson.utmem.edu/CytochromeP450.html) [77]. Transporters were classified based on the Transport Classification Database (http://www.tcdb.org/tcdb/) [78]. G-protein-coupled receptors were selected from the best hits to GPCRDB sequences (http://www.gpcr.org/7 tm/) [79]. Transcription factors were classified by Blastp searches against the Fungal Transcription Factor Database (http://ftfd.snu.ac.kr/) [80]. Protein kinases were classified by Blastp analysis against the KinBase database (http://kinase.com/) with a cutoff E value of 1e^−15^ [81]. Carbohydrate-active enzymes (CAZymes) were classified by local Blastp searches against a library of catalytic and carbohydrate-binding module enzymes constructed from the CAZymes database (http://www.cazy.org/) [82]. To further study the distribution of lignin-modifying enzymes (LME) in fungal genomes, sequences of LME were extracted from the NCBI database. To build the LME database, our custom program compiled with perl was used to choose sequences based on the Blast results (Blastp, cut-off e-value >1e^−50^) from the obtained sequences. LME families were classified by Blastp against the LME database. Multigene families were generated from all the predicted proteins of selected genomes using SCPS tools [83] with default settings (Blastp, cut-off e-value >1e^−30^). The obtained multigene families were then analyzed for evolutionary changes in protein family size (>2) using the CAFE program [84].

### Phylogenomic analysis and molecular clock estimation

Together with *V*. *volvacea*, 14 fungal species assigned mainly to the Basidiomycota (phyla are not italicized) and Ascomycota were used in the phylogenomic analysis. Genomic data for eight species (*Agaricus bisporus*, *Aspergillus niger*, *Ganoderma lucidum*, *Phanerochaete chrysosporium*, *Puccinia graminis*, *Saccharomyces cerevisiae*, *Schizophyllum commune* and *Trichoderma reesei*) were obtained from the Joint Genome Institute (JGI), and for five species (*Coprinopsis cinerea*, *Cryptococcus neoformans*, *Neurospora crassa*, *Rhizopus oryzae*, *Ustilago maydis*) from the Broad Institute. Single-copy orthologous protein sequences from the genomes of 14 species were obtained using our custom perl program. The tandem concatenated sequences consisting of 178 single-copy orthologous sequences from the 14 species were then used to construct a phylogenomic tree using the neighbor-joining method [85] (bootstrap  = 1000, JTT matrix). Timescale estimation of phylogenomic analysis with calibration was implemented in the codeml program [86] of the PAML package (version 4) [70]. The divergence time between species was estimated using PAML [87] by calibrating against the reassessed origin of *U*. *maydis* at 550 million years ago [88]. We used both global and local clock methods [89] to estimate the timescale of species divergence, and linear regression was applied to test the congruence between the global and local clocks.

### Data availability and accession numbers

Data from this Whole Genome Shotgun project have been deposited at DDBJ/EMBL/GenBank (http://www.ncbi.nlm.nih.gov/) under the accession number AMXZ00000000. The version described in this paper is the first version, AMXZ01000000. The transcript data have been deposited at NCBI SRA under the accession number: SRA061941.

## Supporting Information

Figure S1 **Fruiting body of** ***V. volvacea*** **.** (A). Fresh fruiting body. (B). Damaged fruiting body after exposure to 4 °C for 12 hours.
(TIF)

Figure S2 **The number of reductions in the gene families of** ***V. volvacea*** **and other basidiomycetes.** The abbreviation: abis (*Agaricus bisporus*), ccin (*Coprinopsis cinerea*), cneo (*Cryptococcus neoformans*), gluc (*Ganoderma lucidum*), pchr (*Phanerochaete chrysosporium*), scom (*Schizophyllum commune*), umay (*Ustilago maydis*), and vvol (*V. volvacea*).
(TIF)

Figure S3 **Number of gene families in** ***V. volvacea*** **and other basidiomycetes.**
(TIF)

Figure S4 **Distribution of paralogous genes with different levels of amino acid sequence similarity in** ***V. volvacea*** **and other basidiomycetes.**
(TIF)

Figure S5 **Distribution of laccase genes in the genome of** ***V. volvacea*** **and other basidiomycetes.**
(TIF)

Figure S6 **A. Neighbor joining tree of the deduced amino acid sequences of** ***V. volvacea*** **laccase gene.** B. Intron positions within the laccase genes *lac1*-*lac11* define two gene subfamilies. Black bars indicate intron positions. Dotted lines link the same introns.
(TIF)

Figure S7 **Heatmap showing expression levels of genes in starch and sucrose metabolism pathway at 0, 2 and 4 h after exposure to 4°C.**
(TIF)

Figure S8 **Heatmap showing expression levels of genes in glycolysis and gluconeogenesis pathways at 0, 2 and 4 h after exposure to 4°C.**
(TIF)

Figure S9 **Heatmap showing expression levels of genes in unsaturated fatty acid biosynthesis at 0, 2 and 4 h after exposure to 4°C.**
(TIF)

Table S1 ***V. volvacea*** **gene annotation.**
(XLSX)

Table S2 ***V. volvacea*** **gene KEGG analysis.**
(XLSX)

Table S3 ***V. volvacea*** **gene GO analysis.**
(XLSX)

Table S4 ***V. volvacea*** **Pfam analysis.**
(XLSX)

Table S5 **The transfer RNA (tRNA) units in** ***V. volvacea*** **genome.**
(XLSX)

Table S6 **The microsatellite sites in** ***V. volvacea*** **genome.**
(XLSX)

Table S7 **Protease genes in different fungal genomes, arranged by MEROPS family.**
(XLSX)

Table S8 **The number and the relative ration of CYPs in basidiomycetes.**
(XLSX)

Table S9 **Cytochrome P450 (CYP) genes encoded in** ***V. volvacea*** **,** ***C. cinerea*** **,** ***A. bisporus*** **and** ***S. commune*** **genomes, arranged by CYP family.**
(XLSX)

Table S10 **Carbohydrate-degrading enzymes in** ***V. volvacea*** **and other basidiomycetes.**
(XLSX)

Table S11 **Comparative analysis of the number of CAZy families related to plant polysaccharide degradation in** ***V. volvacea*** **and other basidiomycetes.**
(XLSX)

Table S12 **The number of genes encoding the lignin oxidative enzymes in** ***V. volvacea*** **and other basidiomycetes.**
(XLSX)

Table S13 **The basic information of eleven laccase genes in** ***V. volvacea*** **genome.**
(XLSX)

Table S14 **The expressed genes in mycelia of** ***V. volvacea*** **during 4 °C exposure at 0 h, 2 h and 4 h.**
(XLSX)

Table S15 **The up-expression and down-expression genes in** ***V. volvacea*** **during 4 °C exprosure at 2 h and 4 h.**
(XLSX)

Table S16 **The expression of genes encoding the heat shock protein in** ***V. volvacea*** **during 4 °C exposure at 2 h and 4 h.**
(XLSX)

Table S17 **The genes expression of starch and sucrose metabolism pathway in** ***V. volvacea*** **during 4 °C exposure at 2 h and 4 h.**
(XLSX)

Table S18 **The genes expression of glycolysis and gluconeogenesis metabolism pathway in** ***V. volvacea*** **during 4 °C exposure at 2 h and 4 h.**
(XLSX)

Table S19 **The genes expression of unsaturated fatty acid biosynthesis pathway in** ***V. volvacea*** **during 4 °C exposure at 2 h and 4 h.**
(XLSX)
