# Supplementary figures and images for: Sequencing and Comparative Analysis of the Straw Mushroom (Volvariella volvacea) Genome
Source: PLoS One. 2013 Mar 19;8(3):e58294. doi: 10.1371/journal.pone.0058294 (PMC3602538; doi:10.1371/journal.pone.0058294)

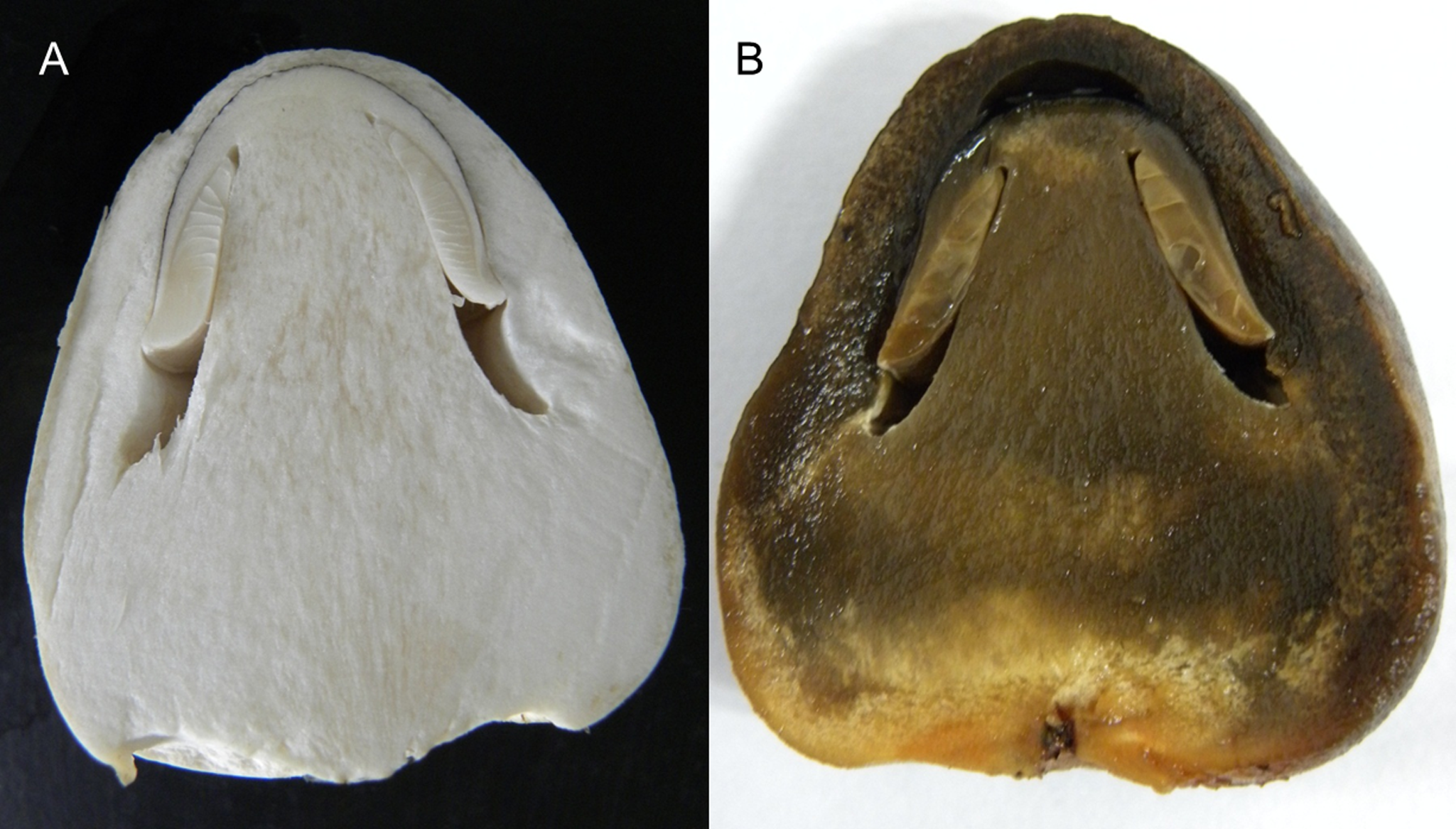

Supplement: Figure S1 — Fruiting body of V. volvacea . (A). Fresh fruiting body. (B). Damaged fruiting body after exposure to 4 °C for 12 hours. (TIF) [file pone.0058294.s001.tif]

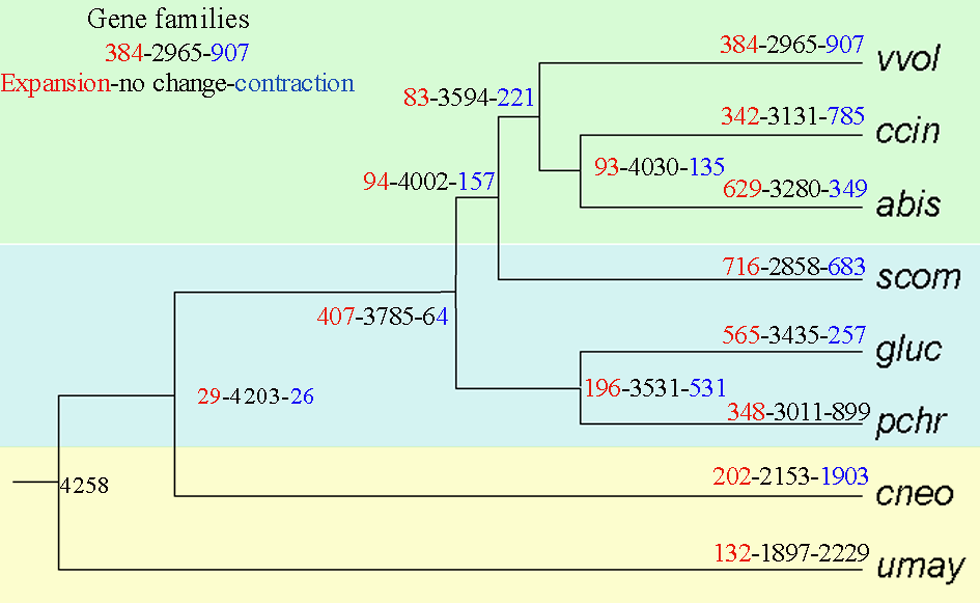

Supplement: Figure S2 — The number of reductions in the gene families of V. volvacea and other basidiomycetes. The abbreviation: abis (Agaricus bisporus), ccin (Coprinopsis cinerea), cneo (Cryptococcus neoformans), gluc (Ganoderma lucidum), pchr (Phanerochaete chrysosporium), scom (Schizophyllum commune), umay (Ustilago maydis), and vvol (V. volvacea). (TIF) [file pone.0058294.s002.tif]

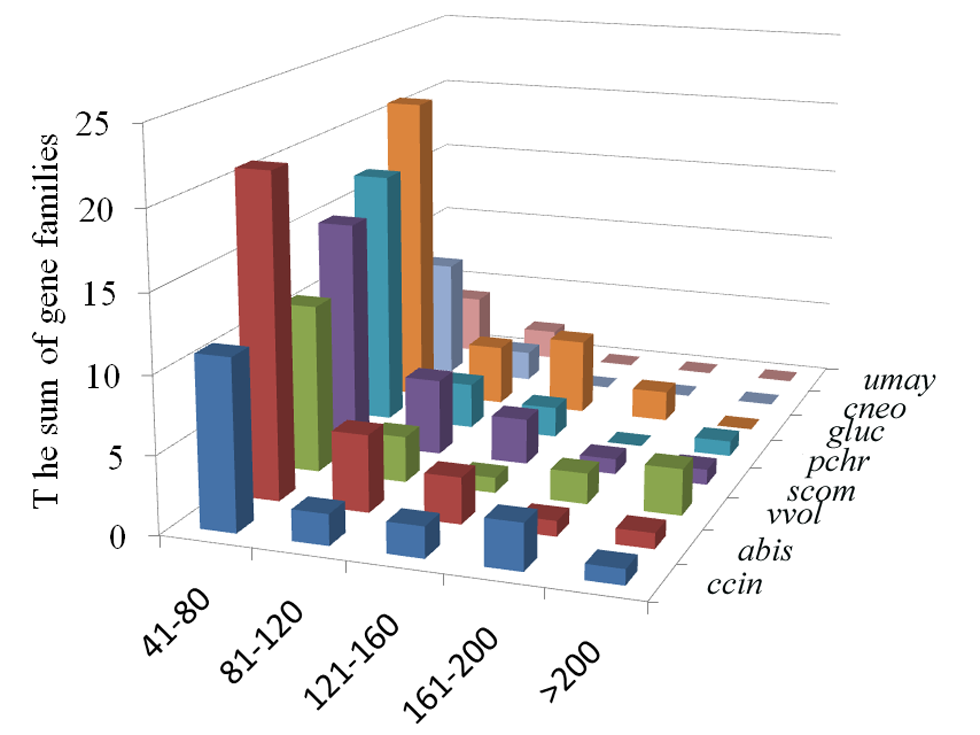

Supplement: Figure S3 — Number of gene families in V. volvacea and other basidiomycetes. (TIF) [file pone.0058294.s003.tif]

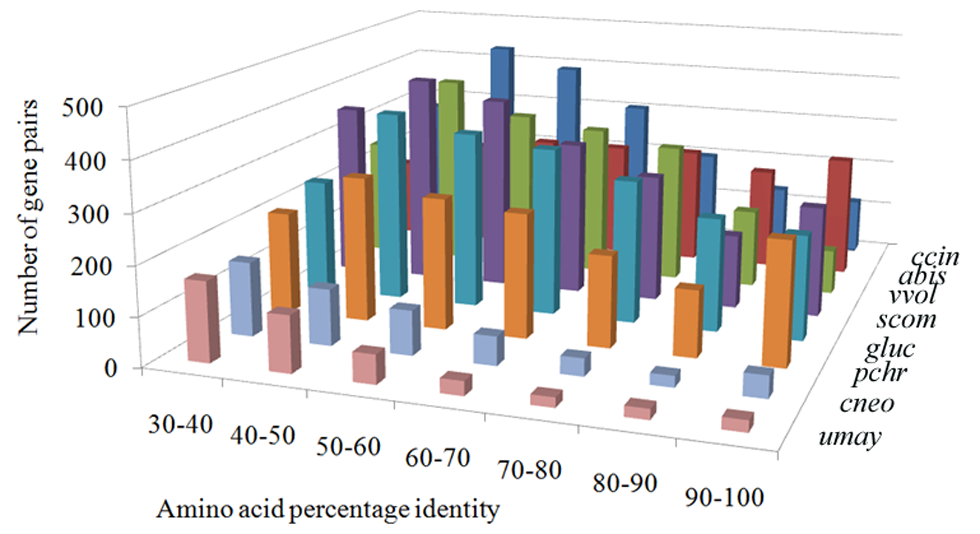

Supplement: Figure S4 — Distribution of paralogous genes with different levels of amino acid sequence similarity in V. volvacea and other basidiomycetes. (TIF) [file pone.0058294.s004.tif]

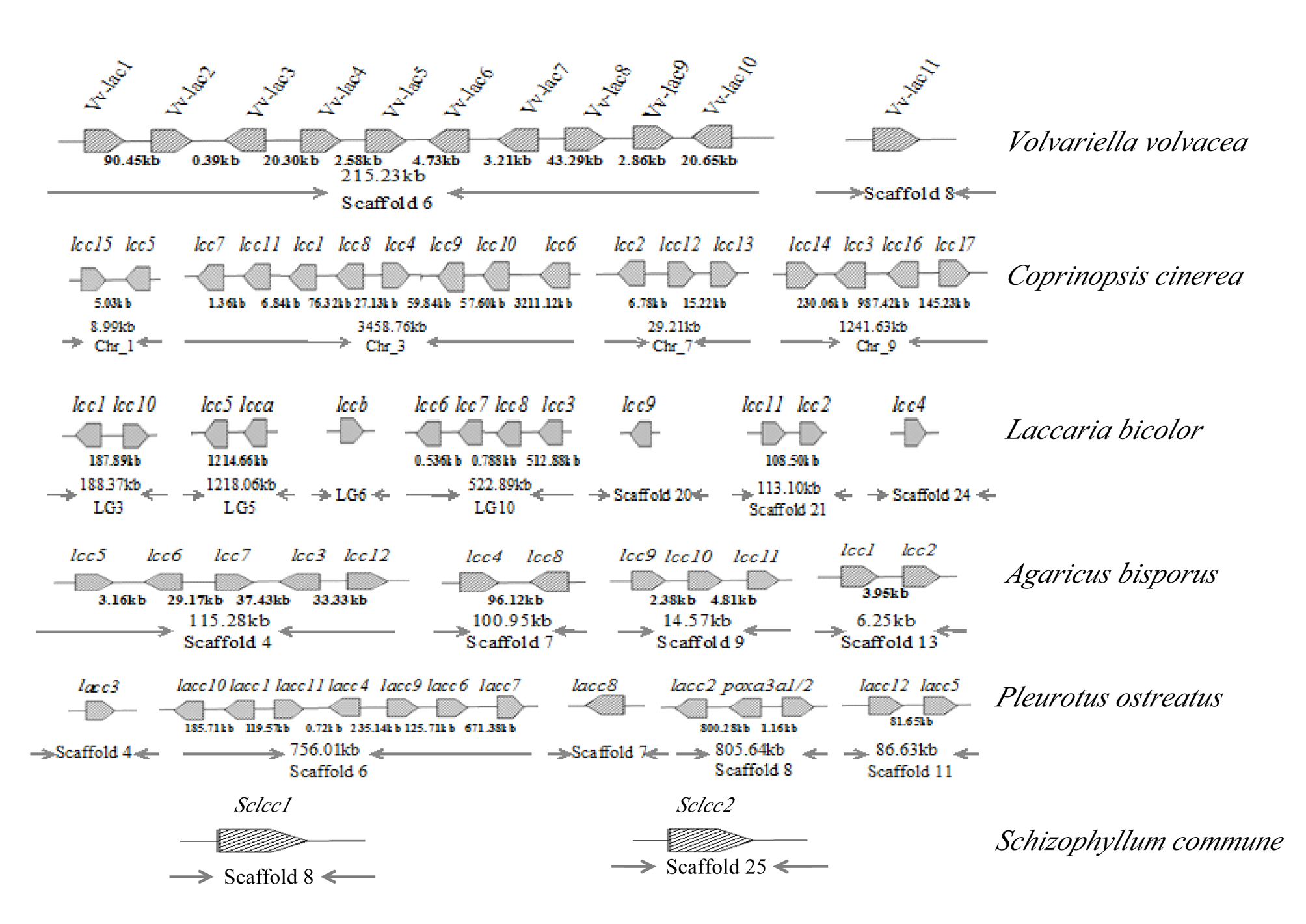

Supplement: Figure S5 — Distribution of laccase genes in the genome of V. volvacea and other basidiomycetes. (TIF) [file pone.0058294.s005.tif]

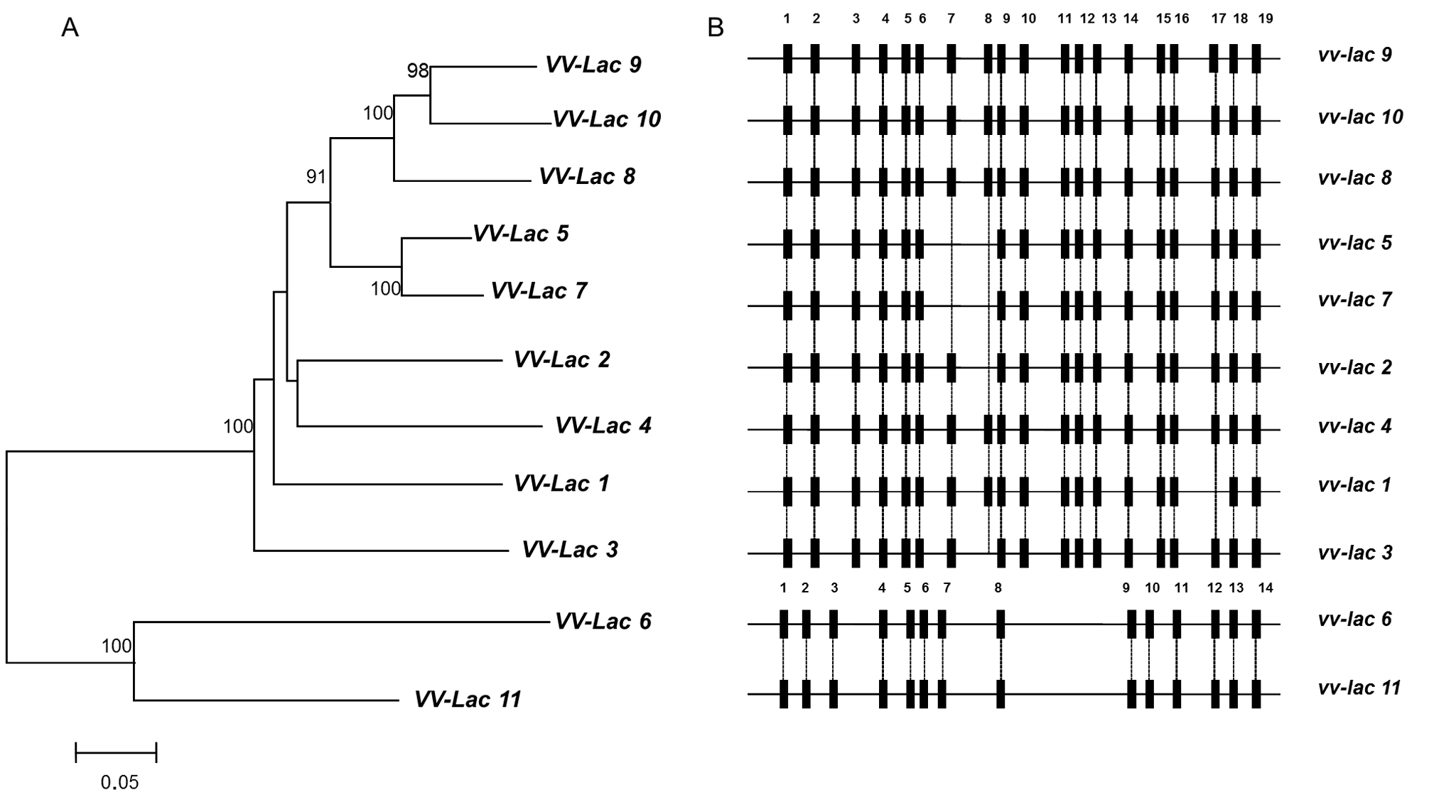

Supplement: Figure S6 — A. Neighbor joining tree of the deduced amino acid sequences of V. volvacea laccase gene. B. Intron positions within the laccase genes lac1-lac11 define two gene subfamilies. Black bars indicate intron positions. Dotted lines link the same introns. (TIF) [file pone.0058294.s006.tif]

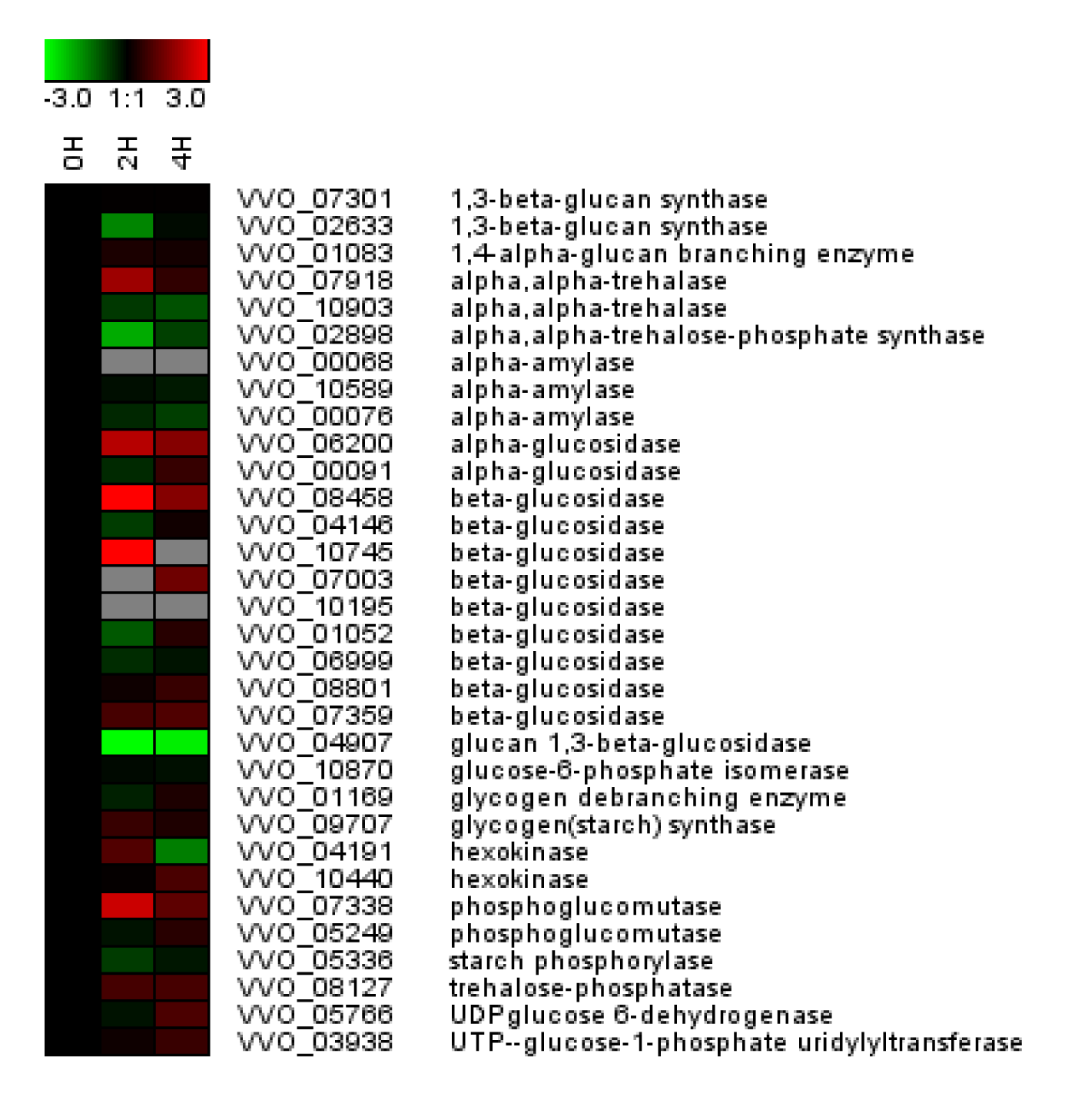

Supplement: Figure S7 — Heatmap showing expression levels of genes in starch and sucrose metabolism pathway at 0, 2 and 4 h after exposure to 4°C. (TIF) [file pone.0058294.s007.tif]

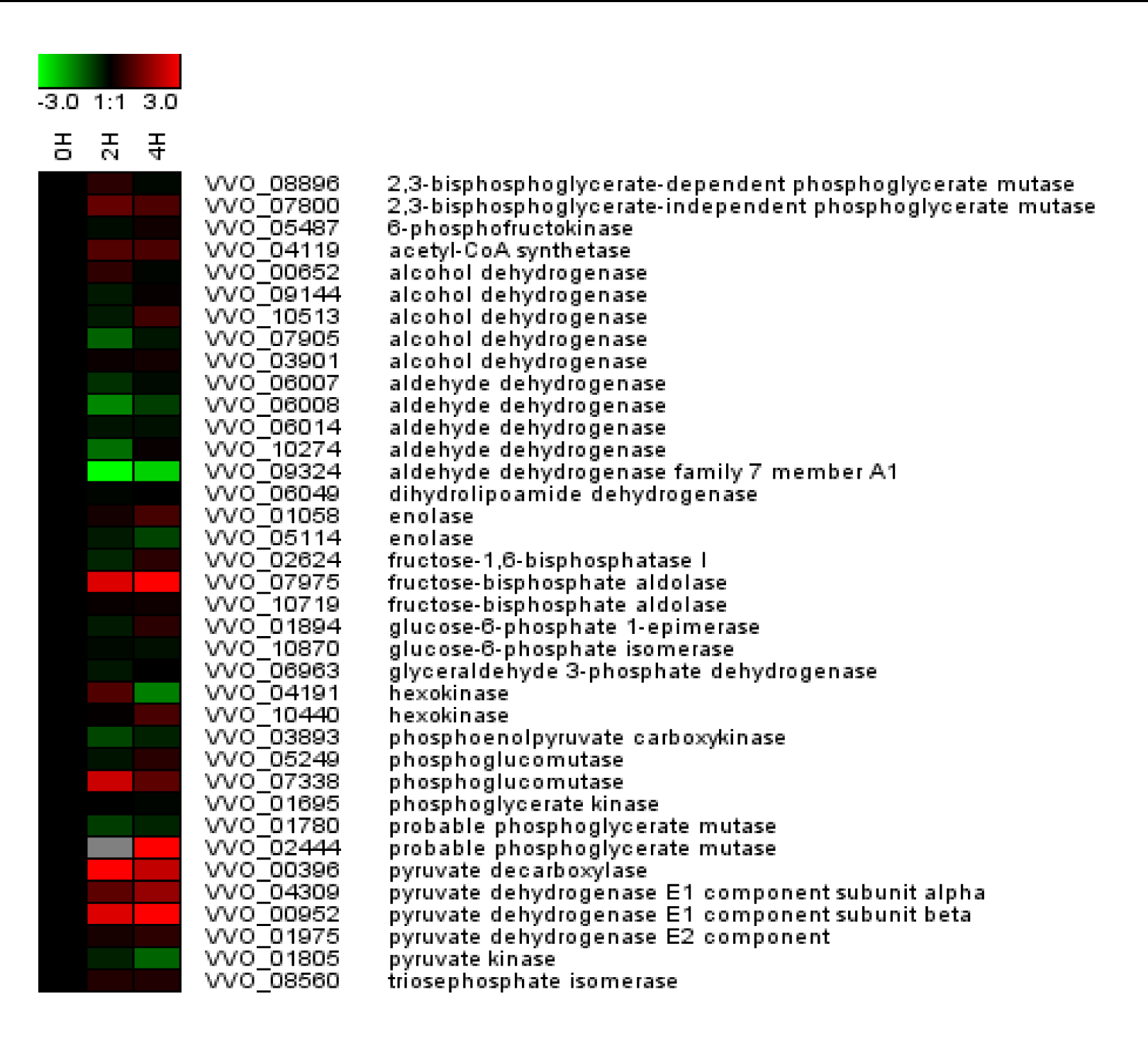

Supplement: Figure S8 — Heatmap showing expression levels of genes in glycolysis and gluconeogenesis pathways at 0, 2 and 4 h after exposure to 4°C. (TIF) [file pone.0058294.s008.tif]

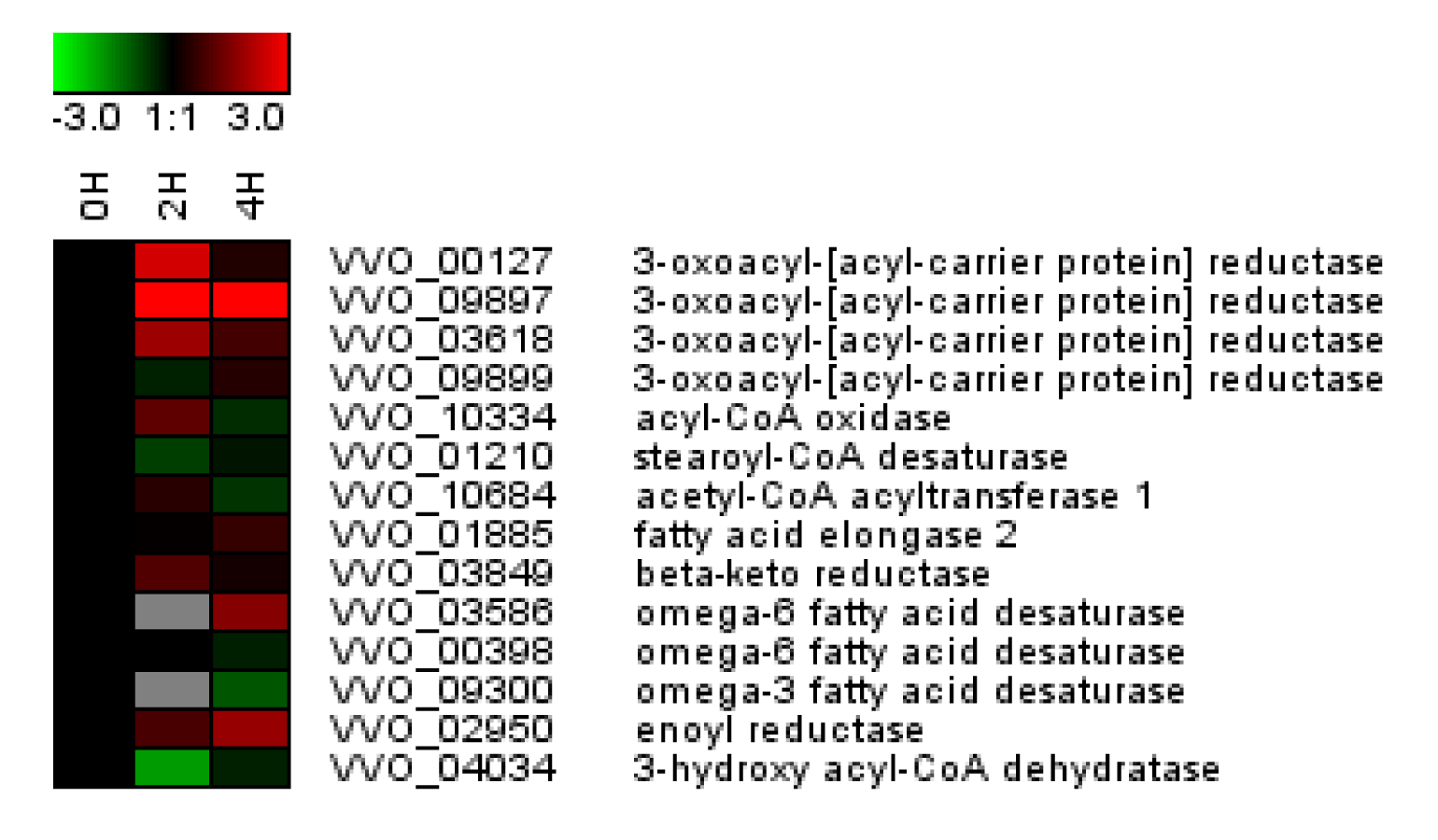

Supplement: Figure S9 — Heatmap showing expression levels of genes in unsaturated fatty acid biosynthesis at 0, 2 and 4 h after exposure to 4°C. (TIF) [file pone.0058294.s009.tif]
